# *Lactobacillus paracasei* Comparative Genomics: Towards Species Pan-Genome Definition and Exploitation of Diversity

**DOI:** 10.1371/journal.pone.0068731

**Published:** 2013-07-19

**Authors:** Tamara Smokvina, Michiel Wels, Justyna Polka, Christian Chervaux, Sylvain Brisse, Jos Boekhorst, Johan E. T. van Hylckama Vlieg, Roland J. Siezen

**Affiliations:** 1 Danone Research, Palaiseau, France; 2 Institut Pasteur, Genotyping of Pathogens and Public Health platform, Paris, France; 3 NIZO food research, Ede, The Netherlands; 4 Centre for Molecular and Biomolecular Informatics (CMBI), Radboud University Medical Centre, Nijmegen, The Netherlands; 5 Netherlands Bioinformatics Centre (NBIC), Nijmegen, The Netherlands; 6 Kluyver Center for Genomics of Industrial Fermentation, Delft, The Netherlands; 7 Microbial Bioinformatics, Ede, The Netherlands; Baylor College of Medicine, United States of America

## Abstract

*Lactobacillus paracasei* is a member of the normal human and animal gut microbiota and is used extensively in the food industry in starter cultures for dairy products or as probiotics. With the development of low-cost, high-throughput sequencing techniques it has become feasible to sequence many different strains of one species and to determine its “pan-genome”. We have sequenced the genomes of 34 different *L. paracasei* strains, and performed a comparative genomics analysis. We analysed genome synteny and content, focussing on the pan-genome, core genome and variable genome. Each genome was shown to contain around 2800–3100 protein-coding genes, and comparative analysis identified over 4200 ortholog groups that comprise the pan-genome of this species, of which about 1800 ortholog groups make up the conserved core. Several factors previously associated with host-microbe interactions such as pili, cell-envelope proteinase, hydrolases p40 and p75 or the capacity to produce short branched-chain fatty acids (*bkd* operon) are part of the *L. paracasei* core genome present in all analysed strains. The variome consists mainly of hypothetical proteins, phages, plasmids, transposon/conjugative elements, and known functions such as sugar metabolism, cell-surface proteins, transporters, CRISPR-associated proteins, and EPS biosynthesis proteins. An enormous variety and variability of sugar utilization gene cassettes were identified, with each strain harbouring between 25–53 cassettes, reflecting the high adaptability of *L. paracasei* to different niches. A phylogenomic tree was constructed based on total genome contents, and together with an analysis of horizontal gene transfer events we conclude that evolution of these *L. paracasei* strains is complex and not always related to niche adaptation. The results of this genome content comparison was used, together with high-throughput growth experiments on various carbohydrates, to perform gene-trait matching analysis, in order to link the distribution pattern of a specific phenotype to the presence/absence of specific sets of genes.

## Introduction

Lactic acid bacteria (LAB) are Gram-positive bacteria that produce lactic acid as their major fermentation end product, and are often involved in food and feed fermentations [1], [2]. The most diverse genus of LAB is *Lactobacillus*, which encompasses species found mainly in dairy products (e.g., *Lactobacillus delbrueckii* ssp. *bulgaricus* and *L. helveticus*), species commonly found in human and animal gastrointestinal tracts (e.g., *Lactobacillus acidophilus* and *Lactobacillus gasseri*), and species with remarkable adaptability to diverse habitats (e.g., *Lactobacillus plantarum, L. pentosus, L. brevis* and *L. paracasei*) [3].

*Lactobacillus paracasei* is a member of the normal human and animal gut microbiota and is used extensively in the food industry in starter cultures for dairy products and also as bacteria with probiotic features [4], [5]. The nomenclature of *Lactobacillus casei* and *paracasei* has been a matter of extensive debate [6], [7], [8]. The majority of the strains designated as either *L. casei* or *L. paracasei* subsp. *paracasei* in literature are members of the same species which should normally be named *Lactobacillus paracasei* subsp. *paracasei* following the current valid nomenclature [9], [10]. In this paper we will use both *L.casei* and *L.paracasei* since many publications refer to both species names.

Several *L. casei/paracasei* strains used in dairy products were previously clinically studied and their beneficial effects assessed [11], [12], [13], [14], [15], [16], [17]. Strains of this species have also been isolated from a variety of fermented artisanal products such as fermented milk, cheese, sourdough bread starter, and fermented vegetables, as well as from plants. Robust genotyping methods have been developed for strain tracking, collection management and population biology research. For this study we used a highly diverse collection of *L. casei/paracasei* strains isolated from different ecological niches such as fermented milk or cereal products, human and animal gut or plants. Previously, the genetic diversity and strain evolution has been assessed for 52 strains of *L. casei/paracasei* from this collection using multilocus sequence typing (MLST) based on sequence variations in 7 housekeeping genes, and revealed 31 different sequence types, with one dominating sequence type (ST1) present in many dairy strains [13]. A similar study has been done for 40 *L. cas*ei strains from a different collection where 36 sequence types were identified [18], [19]. The relatively high number of sequence types can be due to a combination of factors, including a high rate of nucleotide substitution, which generates novel alleles, and frequent homologous recombination, leading to novel combinations of alleles at individual genes. The latter phenomenon was demonstrated in *L. casei* [13] as nucleotides are approximately four times more likely to change by homologous recombination than by mutation, which is comparable with the situation in *E. coli*.

Comparative genome hybridization has been used to analyse genomic diversity in several species of lactic acid bacteria, including *L. casei* [19]. While this method provides a useful first insight into diversity in whole genome content of multiple strains, there are severe limitations since (i) CGH analysis is limited to genes that are present in the reference genomes used for design of arrays, and (ii) genes which show poor hybridization to the array will be missed [20]. With the development of low-cost, high-throughput sequencing techniques it has become feasible to sequence full genomes of many different strains of one species to determine genomic diversity and the species “pan-genome” [21], [22], [23]. Some examples are *Streptococcus agalactiae* [23], *Streptococcus pneumoniae* [24], *Enterococcus faecium* [25], *Escherichia coli* [26], and *Salmonella enterica* [27], but to date this approach has only very recently been reported for a single LAB species, i.e. *Oenococcus oeni* [28].

Complete genome sequences of five *L. casei/paracasei* strains are publicly available [29], [30], [31], [32], [33], as well as draft genomes of two additional strains; plasmids were identified in four of these genomes (**Table S1**). The genomes are all about 2.9–3.0 Mb in size, with a GC content of 46.2–46.6%, and they are predicted to encode 2800–3100 proteins. Better knowledge of the variability and specificities of this industrially important species could contribute to the understanding of its capacity to adapt to different environments, and its particularities in the interaction with the host. To this end, we obtained draft genome sequences of 34 selected strains.

Specific focus was placed on differences in encoded extracellular components of lactobacilli which are putatively involved in host–cell interactions and potentially affecting host health. These components comprise a variety of cell envelope-bound or secreted proteins and polysaccharides (EPS). *Lactobacillus rhamnosus* GG has LPxTG-anchored pilin proteins (encoded by *spaBCA* and *spaDEF* genes) and a pilin-specific sortase [34], and it has been demonstrated that these pili can play a role in mucus binding [35]. *Lactobacillus paracasei* and *Lactobacillus rhamnosus* strains produce the cell-surface associated cell-wall hydrolases Msp2/p40 and Msp1/p75 [36], [37], which display anti-apoptotic and cell-protective effects on human epithelial cells [34], [38], [39]. They have been shown to bind to mucin, collagen and cultured epithelial cells [38]. So-called “collagen-binding” proteins are large extracellular peptidoglycan-bound proteins with CnaB domains which may be involved in adhesion to other cells and host tissues [40]. Cell-surface complex proteins encoded by *cscABCD* genes are found in many gram-positive bacteria, and have been proposed to be involved in plant polysaccharide degradation [41]. The CscB and CscC proteins have a C-terminal WxL domain which has been shown to be involved in cell-wall binding in *Enterococcus faecalis* [42]. Extracellular cell-envelope-bound subtilisin-like serine proteinases of lactic acid bacteria, also called lactocepins or subtilases [43], are important for bacterial growth on proteinaceous substrates, like milk caseins in dairy fermentations [44], [45]. Recently, the *Lactobacillus paracasei* lactocepin PrtP was also shown to selectively degrade secreted, cell-associated, and tissue-distributed IP-10 and other pro-inflammatory chemokines, resulting in significantly reduced inflammation [46]. Extracellular polysaccharides may also play a role in adhesion and/or biofilm formation [47], [48].

The availability of these genome content data allows the definition of gene sets that are common to all *L. paracasei* strains. On the other hand it opens the possibility to correlate gene variation among different strains to the presence (or absence) of phenotypic traits, allowing further in-depth mechanistic insight and the development of genetic biomarkers for detection of interesting phenotypic traits. In this study, we performed growth experiments of all strains on a large number of carbohydrates, and used the novel gene-trait matching tool Phenolink [49], which is based on Random Forest algorithms, to find correlations between genotypes and phenotypes. This tool has recently been used successfully for gene-trait matching of multiple strains of *Lactobacillus plantarum* [49] and *Lactococcus lactis* [50].

## Materials and Methods

### Strain selection and DNA isolation

A complete list of the selected *L. paracasei* strains and their origin can be found in **Table 1**. These strains were selected based on our previous MLST analysis of multiple strains and AFLP genotyping analysis (data not shown) to represent the most genetically diverse set of known *L. paracasei* strains [13]. Strains with a CNCM code have been deposited in the CNCM public library (Institute Pasteur, Paris, France). For DNA preparation, 2 ml of overnight culture was pelleted, washed and resuspended in TES buffer (N-[tris(hydroxymethyl)methyl]-2-aminoethanesulfonic acid). Cell lysis was performed with lysozyme (360 mg/ml) and mutanolysin (140 U /ml) during 2 h at 37°C, then 300 µl water was added and 80 µl of 20% SDS solution. The DNA extraction was done using phenol/chloroform (3×). The DNA was precipitated with isopropanol and washed with 70% ethanol. RNAse treatment was performed using 100 µg/ml RNAse (Sigma) during 1 hour at 37°C.

**Table 1:** Overview of *Lactobacillus paracasei* strains and properties.

					CRISPR system				EPS biosynthesis cluster					
strain code	other strain codes	source	year of isolation	(ML)ST type	Lcas1 CRISPR**type	Lcas1	Lcas2	EPS-1	EPS-2	EPS-3A		EPS- 3B^#^	EPS-4	
**sequenced strains**														
Lpp7		*commercial dairy product*	1982						Y			9		
Lpp14		*artisanal dairy product*	1989					Y		Y		15		
Lpp17	D640	*artisanal dairy product*	1987	14	3	Y			Y	Y		4		
Lpp22	D645	*commercial dairy product*	1987	16	0				Y			4		
CNCM I–4648	D647	*artisanal dairy product*	1988	21	0							7		
Lpp37	D657, ATCC27092	*commercial dairy product*	1994	1	1	Y		Y		Y		13		
Lpp41	D661	*commercial dairy product*	1995	17	0				Y			7		
Lpp43	D662	*commercial dairy product*	1995	9	5	Y				Y		13		
Lpp74		*artisanal dairy product*	2000				Y		Y			7		
Lpp120	D695	*artisanal dairy product*	2003	29	0			Y		Y		15		
Lpp122	D697	*artisanal dairy product*	2003	18	0		Y					10		
Lpp123	D693	*artisanal dairy product*	2003	28	7	Y			Y			7		
CNCM I–4649		*artisanal dairy product*	2003		?	Y		Y		Y		6		
Lpp125	D698	*artisanal dairy product*	2003	6	0			Y		Y		12		
Lpp126	D699	*artisanal dairy product*	2003	30	9	Y		Y		Y		9		
Lpp226		*artisanal dairy product*	2009					Y		Y		14		
Lpp219		*human faeces*	2008		13	Y			Y	Y		3		
Lpp221		*human faeces*	2008		?	Y	Y			Y		12		
Lpp223		*human faeces*	2008		1/2	Y		Y		Y		12		
Lpp225		*animal faeces*	2009		10	Y		Y		Y		5	Y	
Lpp227		*human clinical isolate*	2008		12	Y			Y	Y		12		
Lpp228		*human saliva*	2008					Y		Y		14		
Lpp229	ATCC4009	*clinical isolate*	?					Y		Y		8		
Lpp230	ATCC11582	*human saliva*	?						Y	Y		7		
Lpp46	D664, DSM2649	*plant*	1996	11	4	Y			Y	Y		17		
Lpp48		*fermented cereal product*	1996	23	?	Y		Y		Y		4		
Lpp49	D667	*plant (cereals)*	1996	24	0			Y		Y		14		
Lpp70		*fermented cereal product*	1999							Y		14		
Lpp71	D679	*fermented cereal product*	1999	19	0							8		
CNCM I–4270	D685	*fermented cereal product*	2000	26	0				Y	Y		10		
CNCM I 2877	D686	*plant (cereals)*	2000	27	6	Y				Y		9		
Lpp189		*plant (cereals)*	2005					Y		Y		14		
Lpl7		*plant (cereals)*	?		11	Y			Y	Y		16		
Lpl14		*plant (cereals)*	1996		11	Y			Y	Y		16		
**reference strains**														
ATCC334	D671	*cheese*	?	25	0		Y	Y		Y	13			
BL23	D692	*unknown*	?	1	1	Y		Y		Y	13			
Zhang		*fermented mare's milk*	?		1	Y			Y	Y	16			

Y  = present ; #  = number of OGs; ?  = unknown.

### Genome sequencing and annotation

Draft genome sequences of 34 *L. paracasei* strains were obtained (GATC Biotech, Germany) using 454 GS FLX sequencing at different sequence qualities and coverage ranging from 6–32x (see complete sequencing statistics in **Table S2**). In addition, genome sequences of three publicly available *L. casei* strains were used for comparison, i.e. ATCC 334 [31], BL23 [32] and Zhang [33] (**Table S1**).

We selected 10 genomes with the highest coverage and/or assembly for a complete *de novo* RAST pipeline annotation [51]. The remaining genome sequences were subjected to multiple open-reading frame (ORF) calling tools, i.e. Genemark [52], Glimmer [53], ZCurve [54] and Prodigal [52], [53], [54], [55]. From these tools a majority vote system was used to decide presence or absence of a coding sequence. In all cases, only limited overlap (max 100 bp) was allowed between different predicted coding sequences. In case of a larger overlap, the smaller of the two ORFs was discarded. Further annotation of these genomes was performed through orthology analysis (see below). In case of genes being found exclusively within the set of non-annotated strains, manual annotation of the gene function was performed using BLASTP and InterproScan to come to a function prediction, when necessary.

Genome mapping to reference genomes was performed using the protein coding sequences (CDS) of the genomes and comparing them using Inparanoid [56]. Contigs were organised in their most likely order on the basis of the mean start/stop coordinate of the Inparanoid hit on the reference genomes. Pseudo-assemblies for all genomes were created based on the circular reference genomes of *L. casei* ATCC 334 [31], BL23 [32] and Zhang [33].

The 10 draft *L. paracasei* genomes with our detailed annotation have been deposited at DDBJ/EMBL/GenBank as Whole Genome Shotgun Bioprojects PRJNA178446-PRJNA178455. The other 24 draft *L. paracasei* genomes were only deposited as contig nucleotide sequences in Whole Genome Shotgun Bioprojects PRJNA178422–PRJNA178445. All sequences can be found under umbrella BioProject PRJNA183193.

### Comparative genomics/orthology prediction

All protein sequences of the 37 *L. paracasei* genomes were subjected to an orthology prediction using OrthoMCL [57], with default settings. The protein sequences of 183 OGs with exactly one member in each *L. paracasei* genome were aligned using MUSCLE [58]. These alignments were concatenated after which a maximum-likelihood tree was constructed using PHYML [59]. The output from OrthoMCL was parsed, using *ad hoc* Python scripts, into a single gene presence/absence matrix. This presence/absence matrix was fed into Genesis [60] to perform a hierarchical clustering on the data. Annotation was manually improved, using different sources (public strains, previously annotated genomes and RAST) and using tools such as NCBI BLASTP (http://blast.ncbi.nlm.nih.gov/) and Interproscan (http://www.ebi.ac.uk/Tools/pfa/iprscan/), and added to the matrix on the basis of individual genes present in the orthologous group. The CRISPRs Finder tool (http://crispr.u-psud.fr/Server/) was used to search for CRISPR direct repeats and spacers in the 34 sequenced *L. paracasei* strains. Identified CRISPRs were compared with a separate PCR analysis of CRISPR and MLST types (Supporting Information S1: CRISPR analysis and **Figure S1**).

### Plasmid prediction

Contigs that represent plasmids were predicted based on one or preferably more of the following criteria: (1) they do not map to the reference chromosomes, (2) they encode typical plasmid functions, (3) they map to published plasmids, (4) they appear to be circular.

### Growth characteristics

Growth was measured on the sugars galactose, cellobiose, dulcitol, mannitol, sorbose, mannose, saccharose, sorbitol, trehalose, maltose, myoinositol, ribose, xylose, lactose, glucose, galactosamine, Ca-gluconate, melezitose, and melibiose. Each strain was pre-cultured in de Man, Rogosa and Sharpe (MRS) medium supplemented with 1% galactose (to avoid catabolic repression) for 18 h at 37°C; 1% of this pre-culture was used to inoculate 300 µL MRS medium (without glucose) supplemented with the different carbohydrates tested. Cultures were grown for 24h at 37°C in 96-well plates and the OD was measured every 20 minutes after 10 sec shaking. We considered that the strain grows on the tested sugar when OD reaches more than 0.8. The blank is represented by MRS without bacteria.

### Gene trait matching (GTM) and visualization of the GTM data

GTM was performed with Phenolink, a random forest (RF)-based phenotype/genotype matching algorithm [49] http://bamics2.cmbi.ru.nl/websoftware/phenolink/). Phenolink works on the basis of Random Forest classification but with some minor adjustments to make it more suitable for GTM (see Methods). The gene presence/absence matrix and the experimental growth data were used as an input for Phenolink, together with a consensus annotation file of the ortholog groups. Typically, sugar growth data was divided into two classes (growth or no growth). Phenolink automatically performs GTM analyses for all the phenotypes and summarizes these data in a single HTML file that can easily be converted into a MSExcel spreadsheet. These output files use a colour scheme to indicate for each measured phenotype which OGs were important in the GTM (importance >0.005) and if the OG was found under (<25%, red) or overrepresented (>75%, green) among the phenotypic class. In addition to these overview pages, HTML-based result pages per phenotypic test were constructed that allow direct analyses of the distribution of the most important OGs among the tested strains.

## Results

### Genome sequencing and comparison

The draft genome sequences were determined of 34 *L. paracasei* strains isolated from various environments (dairy  = 16 strains, plant  = 10, human/animal  = 8) (**Table 1**). The statistics of sequencing and assembly are summarized in **Table S2**. The sequencing coverage ranged from 6–32x, the numbers of assembled contigs from 71–1355, and the genome sizes from 2.7–3.1 Mb.

All sequenced and annotated genomes, as well as genomes of three published reference strains, were subjected to an orthology prediction using OrthoMCL. The results from this prediction were converted into a gene presence/absence matrix (per strain), and used to analyse the pan-genome, the core genome and the variome (also called dispensable or divergent genome) of *L. paracasei*.

#### The *L. paracasei* pan-genome

**Figure 1:**
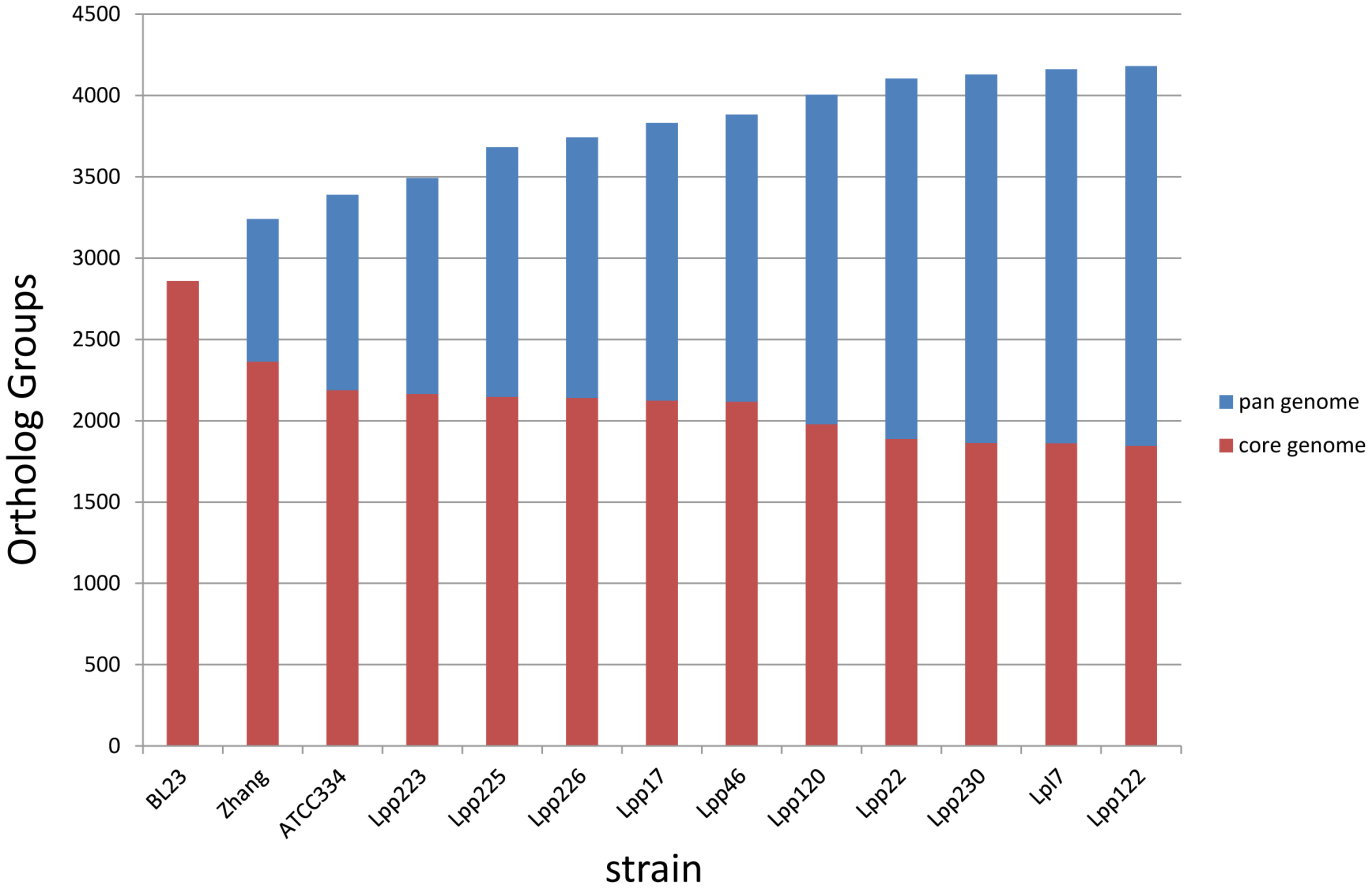
Pan-genome prediction. The number of pan-genome OGs (blue) and core genome OGs (red) is shown as a function of genomes added to the pan-genome. OGs present in only one annotated genome were not included if they appeared to represent gene fragments or overpredicted small genes.

The microbial pan-genome is defined as the full complement of genes in a species, and is typically applied to bacteria and archaea, which can have large variations in gene content among closely related strains [21], [23]. It is the total set of all the genes found in all the strains of a species. A first estimate of the *L. paracasei* pan-genome was calculated using only the 10 RAST-annotated and 3 reference genomes, which have manually curated ORF calling and annotation. We identified a total of about 4200 OGs present in at least two *L paracasei* genomes, of which ∼230 OGs are presumably plasmid-encoded, the “plasmid pan-genome” (see below). **Figure 1** shows the predicted pan-genome size as a function of the number of genomes sequenced. It appears that the pan-genome size is levelling off (at about 4300–4500 genes), as every extra genome adds less new genes. This upper limit may be an overestimate, since some of the draft genomes added have lower coverage, hence poorer ORF prediction and usually overprediction of ORFs due to gene fragments.

#### The *L. paracasei* core genome

**Figure 2:**
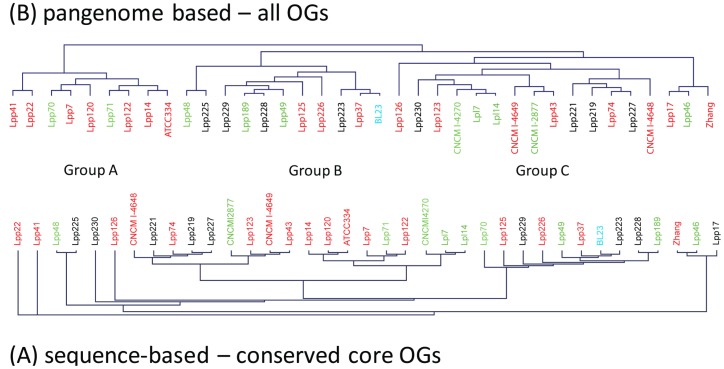
Genetic relatedness of strains. (A) phylogenetic tree based on sequence similarity of 183 orthologous genes present in all strains; (B) pan-genome tree based on total genome content. Red  = dairy strains; green  = plant origin strains; black  = human/animal origin strains; blue  = unknown origin.

The core genome is defined as the orthologous genes (OGs) that are conserved in all strains of a species. **Figure 1** shows that the number of OGs in the core genome decreases as more genome sequences are added, and levels at about 1800 OGs; this may be an underestimate as more core OGs will be missed in low-coverage sequenced genomes. The protein-coding sequences of 183 highly conserved OGs were used to construct a phylogenetic tree (**Figure 2A**), which represents the evolutionary relatedness of the strains. This is essentially MLST using 183 full-length proteins of the core genome, and the tree is highly similar to an MLST tree generated for these strains from seven housekeeping genes (*fusA, ileS, leuS, lepA, pyrG, recA, recG)* used previously [13] (data not shown). However, based on MLST using only these 7 genes, several strains could not be discriminated and were classified as the same sequence type, e.g. Lpp226, Lpp189 and Lpp228 (ST2). As we expect to observe more polymorphisms in 183 genes as compared to 7 genes, all strains that were identical based on 7 genes could be discriminated (but are closely related in the tree) based on the 183 genes. Hence, there are as many sequence types as strains.

There appears to be some correlation between the phylogenetic relatedness and origin of isolation (i.e. dairy, mammal, plant, etc) of these strains, but much less than we anticipated. Even though some of the dairy isolates cluster together phylogenetically, these strains originate from many different countries around the world (data not shown).

**Figure 3:**
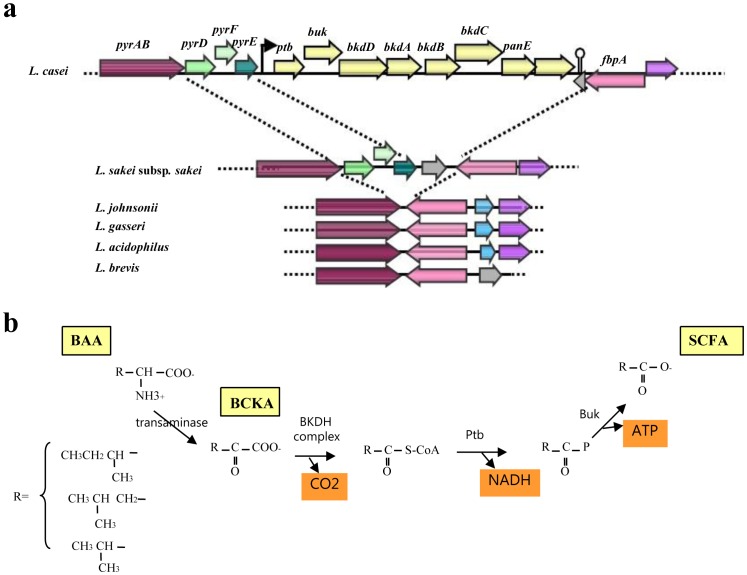
Genetic potential of *L. paracasei* to produce short branched-chain fatty acids. from branched-chain α-keto acids (BCKA). (**a**) Organization of the *bkd* operon in the *L. casei* strains and genetic context in other lactobacilli. The functions encoded by *bkd* genes (yellow) are: Ptb, Phosphate butyryl-transferase; Buk, Butyrate kinase; BkdD, Dihydrolipoamide dehydrogenase; BkdA, 2-oxoisovalerate dehydrogenase a subunit; BkdB, 2-oxoisovalerate dehydrogenase b subunit; BkdC, Lipoamide acyltransferase component of BKDH complex; PanE, Ketopantoate reductase PanE/ApbA. The locus tags of the respective 8 *bkd* genes in the reference genomes are: LSEI_1441–1148 in *L casei* ATCC 334, LCABL_16640–16710 in *L. casei* BL23 and LCAZH_ 1429–1436 in *L. casei* Zhang. The black arrow and the stem-loop indicate a potential promoter and an ρ-independent terminator, respectively. The genetic environment around the *bkd* operon of *L. casei* is conserved among other lactobacilli: orthologous genes are shown by the same colour. PyrAB, Carbamoylphosphate synthase large subunit; PyrD, Dihydroorotate dehydrogenase PyrF; Orotidine-5′-phosphate decarboxylase; PyrE, Orotate phosphoribosyltransferase; FbpA, Fibronectin-binding protein, hypothetical protein LSEI_1438 (**b**) Branched-chain amino acids (BAA) catabolism to fatty acids adapted after [61]. BAA are converted into BCKA via a BAA-amino transferase. The branched-chain α-keto acid dehydrogenase (BKDH) complex is composed of BkdA, BkdB, BkdC and BkdD.

As expected, the core genome includes genes for replication, transcription, translation, central and cell wall metabolism, biosynthesis of most amino acids and metabolism of nucleotides, fatty acids and phospholipids. At least 270 OGs of the core genome are annotated as hypothetical proteins, hence they are totally conserved proteins of as yet unknown function. The core genome also contains at least 15 sugar utilization gene clusters, and a variety of cell-surface components, as discussed in detail below. All *L. paracasei* strains contain an 8-gene cluster which is very similar to the *bkd* operon of *Enterococcus faecalis* [61] involved in the conversion of the branched-chain α-keto acids (BCKA) into branched-chain fatty acids (**Figure 3**).

#### The L. paracasei variome

**Figure 4:**
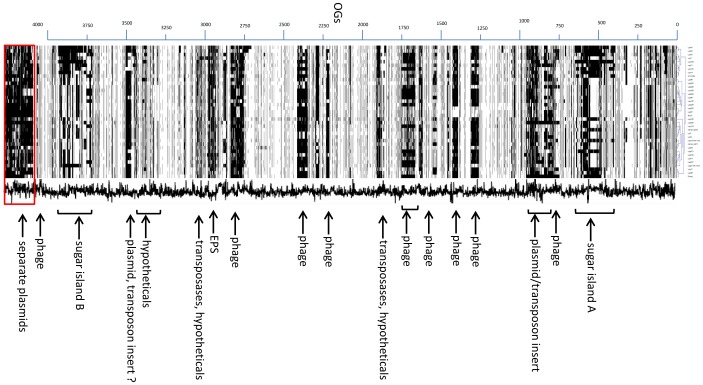
Bar plot of OG presence/absence for the *L. paracasei* strains ordered according to the reference genomes. This figure shows all pan-genome OGs found to be present (white bar) or absent (black bar) on the genomes. The box at the bottom contains OGs on contigs which are presumed plasmids. The pan-genome tree is shown at the top. The scale at the left represents pseudoassembly location relative to the reference genomes. A description of highly variable regions is shown at the right. The GC content is presented in the middle (wavy line), ranging from 30–60% (left to right).

The gene presence/absence matrix was also used for display in Genesis [60], in order to construct bar plots of presence/absence of the different OGs (**Figure 4** **),** and to construct a genome-relatedness or “pan-genome” tree based on total genome content which may correlate better with niche adaptation (**Figure 2B**). Although this pan-genome tree based on the total OG presence/absence matrix is different from the phylogenetic tree (**Figure 2A**), there are many strains that cluster together based both on evolutionary (sequence similarity) relatedness and on niche adaptation.

The pan-genome tree suggests that the strains can be distinguished in 3 main groups A, B, and C. Group A contains mainly strains of dairy origin, while groups B and C contain strains of varying origin. The bar code plot in **Figure 4** shows the pan-genome OGs assembled according to their order in the three circular reference genomes, starting with the entire chromosome, and ending with known and putative plasmids. This pan-genome assembly clearly shows that there are highly constant regions in the 37 genomes, but also highly variable regions which may relate to niche adaptation.

Below we describe the functions encoded in the major variable regions, and their distribution in the *L. paracasei* strains studied.

### Plasmids and the plasmid pan-genome

Many unique functions of lactic acid bacteria have been shown to be encoded on mobile elements such as plasmids and transposons. These functions can include EPS biosynthesis, bacteriocin biosynthesis, proteolysis and flavor formation, sugar metabolism (e.g. lactose), heavy metal resistance, etc. [62], [63], [64], [65].

Contigs representing plasmids were predicted based on several criteria (see Methods), and the main putative plasmids are listed in **Table S3**. This plasmid pan-genome contains about 230 different OGs, and the main encoded functions are listed in **Table S4**. These functions are very similar to those found on plasmids of other LAB, such as *Lactococcus lactis* [66] which is known for its versatility of functions encoded on plasmids that contribute to the production of flavour compounds and to lactose utilization in dairy fermentations. However, the functions encoded on *L. paracasei* plasmids are more limited, and do not appear to include flavour formation, lactose utilization or bacteriocin production. As with most plasmids of LAB, the fraction of hypothetical proteins of unknown function encoded on *L. paracasei* plasmids is rather high compared to those on *L. paracasei* chromosomes. The plasmids can be classified into different groups based on sequence similarity to each other. Several strains contain plasmids larger than 20 kb, as do the reference genomes. Smaller contigs may represent fragments of larger plasmids. No contigs representing plasmids are apparent in strains Lpp37, Lpp230, CNCM I4649, and in the plasmid-free reference genome of strain BL23.

### Horizontal gene transfer

A large highly variable region (∼180 OGs) resembles an inserted plasmid, transposon or integrative conjugative element (ICE) [67], [68] in the pangenome assembly, based on plasmid/transposon-encoded functions, flanking a prophage region with an integrase gene (**Figure 4**; OG region 780–960); the main encoded functions of this transposon region are listed in **Table S4**. This insert region has a much larger variety of functions than the plasmids, in particular a set of transport functions. In some strains this insert region is clearly contiguous with chromosomal genes, and in other strains this region appears on a separate contig(s), putatively a plasmid/transposon, or both; this differs per strain, as does the OG content of this insert. Only strains Lpp230 and Zhang lack this entire insert/plasmid region, including the flanking prophage region. The large majority of genes in this insert region have best Blast hits with 90–100% sequence identity to very related bacteria, i.e. *L. casei/paracasei, L. rhamnosus, L. zeae*, and a GC content very similar to the *L. paracasei* genome. Many genes are similar to those on the published *L. paracasei* plasmids (**Table S1**) and/or the 64-kb plasmid pLC1 of *L. rhamnosus* Lc705 [34]. One exception is a 7-gene cluster, present only in the highly related plant-derived *L. paracasei* strains Lpl7 and Lpl14, that has 97–99% identity to an orthologous cluster in *Oenococcus oeni*, a plant-associated lactic acid bacterium, suggesting that this gene cluster has been acquired recently by the ancestor of Lpl7 and Lpl14. However, it has not been acquired from *O. oeni*, as this gene cluster only occurs in 2 of the 14 sequenced genomes of *O. oeni* and has a GC content of 55%, which is much higher than the GC contents of *L. paracasei* (46%) and *O. oeni* (38%), and hence it presumably has been acquired by both *L. paracasei* and *O. oeni* from an as yet unknown donor.

Two strains (Lpp226, Lpp41) contain an 11-gene insert which is 99–100% identical to a fragment in *L. plantarum* genomes. Since all sequenced *L. plantarum* genomes and related *L. pentosus* genomes contain this fragment, but only two *L. paracasei* strains, it is plausible that *L. paracasei* has acquired this fragment recently through HGT. The encoded functions include a cobalt/nickel (or cobalamin) ABC transporter and the *larABCE* genes for lactate racemization [69]. The latter gene locus is responsible for racemization of L-lactate to D-lactate which can be used in cell wall biosynthesis, replacing the terminal D-Ala by D-lactate, and thereby conferring vancomycin resistance to the cell.

More examples of HGT are given below in specific sections.

### Sugar islands and metabolism

**Figure 5:**
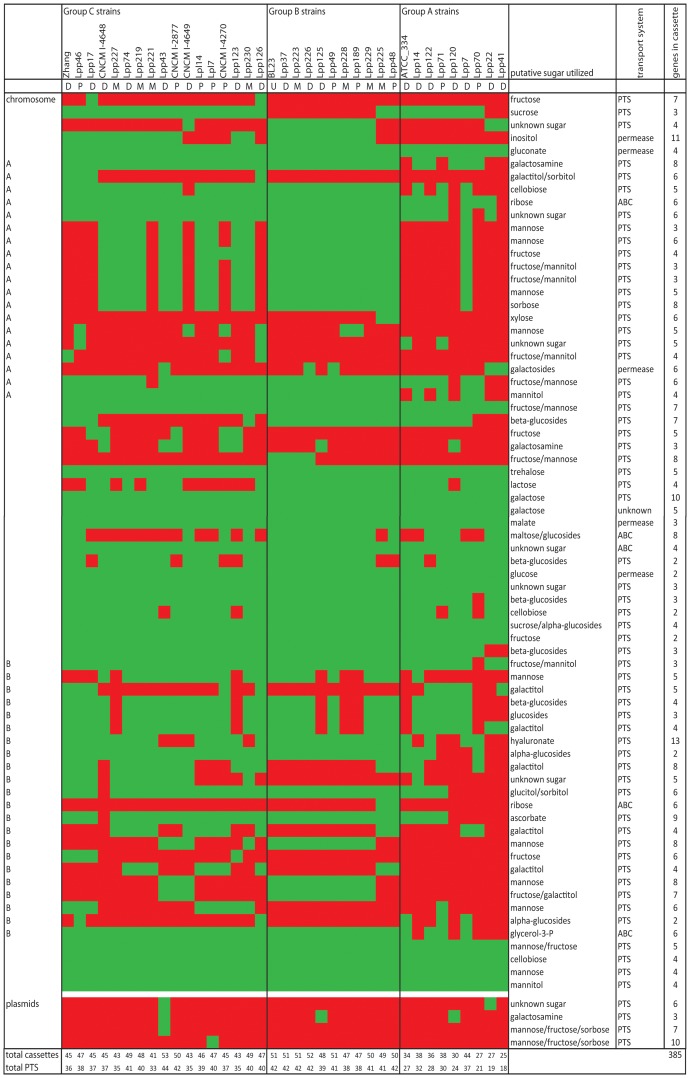
Summary of sugar utilization cassettes. Each row represents the presence (green) or absence (red) of a sugar utilization cassette in the strains listed at the top; D  = dairy origin, P  = plant origin, M  = mammalian origin, U  = unknown origin. The putative sugar(s) utilized, the type of transport system, and the number of genes in each cassette are listed in the last three rows. Group A, group B and group C strains refers to Figure****2B. Chromosomal location: A  = cassette in sugar island A; B  = cassette in sugar island B.

The *L. paracasei* genomes are found to contain numerous sugar utilization cassettes (**Figure 5**) [20], many of which are clustered on two very large sugar islands A and B (**Figure 4**; OG regions 390–640 and 3740–3970, respectively). Sugar island A has about 250 different OGs in the *L. casei* pan-genome, and contains at least 17 PTS-based cassettes, annotated as mannose (5×, i.e.5 different cassettes), fructose/mannitol (5×), beta-glucosides, galactosamine, galactitol/sorbitol, sorbose [70], cellobiose and xylose PTS, in addition to a galactosides permease and a ribose ABC transporter.

Sugar island B of the *L. casei* pan-genome has about 230 different OGs, containing at least 20 PTS-based cassettes, annotated as mannose (4×), galactitol (6×), alpha-glucosides (2×), cellobiose (2×), glucose, fructose, glucitol/sorbitol, ascorbate, and hyaluronate, and additional ABC transporters for ribose and glycerol-3-phosphate.

The distribution of these sugar utilization cassettes is extremely variable throughout the *L. paracasei* genomes, and particularly within the sugar islands A and B. There are a total of 74 sugar utilization cassettes (of which 63 PTS systems), and individual strains contain between 25–53 of these cassettes (**Figure 5**).While some strains contain the majority of the cassettes within sugar islands A and B, other strains lack most of the sugar cassettes (mainly Group A strains in **Figure 2**), and some strains lack nearly the complete sugar islands A and/or B (e.g. strains Lpp22, Lpp41, Lpp70, Lpp120) (see **Figures 4** and **5**). Only 15 of these sugar cassettes are present in all genomes (i.e. belong to the core genome), and they are all located outside the large sugar islands A and B. The differences in overall genome size from 2.7–3.1 Mb are largely due to these differences in presence/absence of sugar islands A and B.

Complete cassettes for utilization of lactose (PTS; 4 genes), maltose (ABC transport; 9 genes), myo-inositol (permease; 11 genes) and hyaluronate-oligosaccharide (PTS; 16 genes) are present in only 20 strains, 21 strains, 19 strains and 28 strains, respectively (**Figure 5**). The lactose PTS cassette appears to be either on the chromosome or on a plasmid (as in strain ATCC 334). In some cases, one sugar cassette appears to replace another, e.g. at one chromosomal location there is either a mannose PTS cassette (22 strains), or a galactitol/sorbitol PTS cassette (8 strains), or neither (8 strains). It is interesting that some specific sugar ABC transport or PTS cassettes are present in only a few strains. Surprisingly, none of these rare occurrences of sugar cassettes appear to correlate with origin of strain isolation.

### Phages

There are about 10 regions with genes encoding typical prophage proteins, containing a total of about 370 OGs, the “phage pan-genome”. The positions of these regions are indicated in **Figure 4** relative to the reference genomes, but in fact some prophages may be inserted at alternative positions, as this cannot be deduced accurately from draft genome sequences. The GC content of most phage regions is considerably lower (38–42%) than the core chromosome (46%) (**Figure 4**).

### CRISPRs

We searched our 34 sequenced genomes for *cas* genes and CRISPR loci [71], [72], [73]. The Lcas1 locus has 4 *cas* genes and is present in reference strains BL23 and Zhang but not in ATCC 334; these *cas* genes were found in 16 other strains (**Table 1**). The Lcas2 locus has 8 *cas* genes in reference strain ATCC 334, and these were found in only 3 other strains (Lpp74, Lpp122, Lpp221). Only strain Lpp221 isolated from human faeces has both the Lcas1 and Las2 loci, whereas 16 strains appear to have neither CRISPR loci (**Table 1**). The Lcas1 locus was found in nearly all Group C strains, in half of Group B strains, and in none of the Group A dairy strains (see **Figure 2B**). The Lcas2 locus is found in only a few unrelated strains, and has probably been acquired more recently by HGT than the Lcas1 locus, as only the Lcas2 is flanked by IS elements and has a GC content of 56–58% (chromosome GC% is 46.5).

All of the Lcas1 CRISPR loci direct repeats and spacers sequences found in the sequenced genomes were in complete agreement with the PCR sequences obtained for those strains (**Table 1** and Supporting Information S1: CRISPR analysis). In many cases, only fragments of the CRISPR loci sequences were found in the assembled genomic contigs, as the numerous direct repeat sequences of 36 nt often prevent proper assembly. Nevertheless, 224 distinct Lcas1 spacers and 25 distinct Lcas2 spacers were identified from PCR and genome sequence analysis together (**Table S5**). For the Lcas1 locus, 13 different CRISPR types differing by the number and identity of CRISPR spacers are distinguished (**Table 1**). These results show that the CRISPR loci contribute to the genomic variation among *L. casei/paracasei* strains.

### Surface-associated and secreted proteins

All extracellular proteins encoded in the reference genomes of *L. casei* ATCC 334 and BL23 have previously been predicted and classified in the LAB Secretome Database [74]. The major surface-associated and secreted proteins identified in the 37 *L. paracasei* genomes are described below and summarized in **Table 2**.

**Table 2:** Main surface-associated and secreted proteins in 37 *L. (para)casei* strains.

protein/complex/cluster	genes	strains present	strains absent	notes	reference
Pili gene cluster					[34],[35]
pilus proteins, pilus-specific sortase	*spaBCA-srtC1*	36	1	absent in strain Lpp125	
pilus proteins, pilus-specific sortase	*srtC2-spaDEF*	37	0		
Csc cell-surface complex CscABCDa					[41], [42]
Csc cluster 1	*cscCDBBA*	37	0		
Csc cluster 2	*cscBADC*	36	1	absent in strain Lpp219	
Csc cluster 3	*cscBDBC*	37	0		
Csc cluster 4	*cscADC*	14	23		
Csc cluster 5	*cacBAAC*	33	4	absent in strains Lpp17, Lpp46, Lpp230, Zhang	
Collagen/fibronection adhesion proteins					[40]
collagen-binding protein CnbA	*cnbA*	37	0	∼2700 AA; 11 CnaB domains; LPSTE anchor^b^	
collagen-binding protein CnbB	*cnbB*	12	25	∼750 AA; 3–4 CnaB domains; MPQTG anchor	
collagen-binding protein CnbC	*cnbC*	7	30	∼900 AA; 4–5 CnaB domains; LPQTG anchor; only plasmid-encoded?	
fibronectin-binding protein FbpA	*fnpA*	37	0	∼576 AA; FbpA and DUF814 domains	
Cell-wall hydrolases					[34], [36], [38], [39], [103]
Msp2/p40	*msp2*	37	0		
Msp1/p75 D-glutamyl-L-lysyl endopeptidase	*msp1*	37	0		
Cell-envelope proteinases				subtilase family serine proteinases	[43], [44],
proteinase PrtP (and its maturase PrtM)	*prtP-prtM*	37	0	∼1900 AA; LPKTA anchor	
proteinase PrtR1, inactive variant	*prtR1*c	37	0	∼1800 AA; LPQMA anchor	
proteinase PrtR2	*prtR2* c	37	0	∼2230 AA; LPPMG anchor	
proteinase PrtR3	*prtR3*	2	35	∼1500 AA; MPQAG anchor; only in strains Lpp120, Lpp122; plasmid-encoded	
Glycoprotein gene cluster				11 genes, also encodes 3 glycosyltransferases	[47], [48]
Ser/Ala-rich glycoprotein		10	27	∼2700 AA; LPQTG anchor	
extracellular protein, unknown function		10	27	∼580 AA; 2 Ig-like and 1 SCP domains	
extracellular protein, unknown function		10	27	∼900 AA; 2 Ig-like domains	
Wss secretion gene cluster		2	35	6 genes, WXG100 secretion system; only in strains Lpp17 and Lpp230	[32], [47], [48], [77], [78], [79], [80], [81], [82], [83]
Extracellular proteins gene cluster				4 genes, only in strains Lpp46, Zhang	
3 extracellular proteins, unknown function		2	35	no LPxTG-type peptidoglycan anchors	

a The *csc* gene cluster can encode different combinations of A, B, C and D subunits [41].

b Refers to LPXTG-type peptidoglycan anchors [104].

c PrtR1 and PrtR2 are encoded on adjacent genes.

All *L. paracasei* strains (except strain Lpp125) have a 7-gene cluster with the pilin-specific *spaBCA* genes, and all have the *spaDEF* genes; this suggests that all *L. paracasei* strains have the potential to synthesize pili which may play a role in mucus binding, adhesion or biofilm formation. A very recent study suggests that upstream insertion of an IS element, as in *L. rhamnosus*, may be required to activate these genes [3]. Similarly, both cell-wall hydrolases Msp2/p40 and Msp1/p75 appear to be encoded in all *L. paracasei* strains analysed in our present study. Several putative collagen-binding proteins are encoded in the *L. paracasei* genomes. A large collagen-binding protein (∼2700 AA) is present in all sequenced *L. paracasei* strains, while a smaller collagen-binding protein (∼750 AA) is not found in the three reference genomes, but is encoded in 12 other *L. paracasei* strains on a putative plasmid that can also be inserted in the chromosome (see above and **Table 2**). Four sequence variants of the latter protein occur, and strains Lpp122 and Lpp41 actually encode two and three different variants, respectively. A third putative collagen-binding protein (∼900 AA) is encoded on plasmid plca36 of *L. casei* Zhang, and also occurs in 6 other strains on putative plasmids, generally as a pseudogene. A single fibronectin-binding protein is encoded in all genomes. The *L. paracasei* genomes have 5 *csc* gene clusters, of which some are not present in all genomes (**Table 2**).

All *L. paracasei* genomes of our present study were found to encode the same three cell-envelope-bound serine proteinases, i.e. the PrtP ortholog (∼1900 AA) and two proteins encoded on adjacent genes representing the serine proteinase PrtR2 (∼2230 AA) and an inactive variant PrtR1 (∼1800 AA); all these subtilases have an LPxTG-type peptidoglycan anchor. Several of the PrtR orthologs appear to be pseudogenes, as has been observed for *L. casei* ATCC 334 [19]. Moreover, the dairy strains Lpp120 and Lpp122 have an extra intact and putatively active PrtR homolog (∼1530 AA), probably encoded on a plasmid (or the large plasmid insert), which has a best BLAST hit (99% amino acid identity) to an ortholog from *Lactobacillus zeae*, a strain isolated from raw bovine milk [75].

An 11-gene cassette, present in 10 *L. paracasei* strains but not in the reference genomes, encodes a very large serine/alanine-rich LPxTG-anchored surface protein, in addition to two other extracellular proteins and three glycosyltransferases. These family 1 glycosyltransferases are known to play a role in O-linked glycosylation of serine-rich cell-surface proteins in streptococci [76], and hence may be involved in glycosylating serine residues of the large Ser/Ala-rich surface protein. The ten *L. paracasei* strains containing this gene cassette are closely related both phylogenetically and in genome content (only in a subset of Group C genomes) (**Figure 2**). As this gene cassette has a GC content of about 46%, it was probably acquired by a common ancestor of these 10 strains through HGT a long time ago. This gene cassette with a Ser/Ala-rich surface-anchored protein is also found in *L. rhamnosus* strains. A 4-gene cassette encoding 3 extracellular proteins with signal peptides is present in *L. casei* Zhang (LCAZH_0540-0543) and only in the very closely related *L. paracasei* strain Lpp46. These extracellular proteins of unknown function do not have LPxTG-type peptidoglycan anchors, and are not homologous to any proteins in the NCBI database. As this locus has a GC content of 39.4% this cassette was probably acquired through HGT by the direct ancestor of these 2 related strains.

Two strains (Lpp17 and Lpp230) are found to contain a 6-gene WXG100 secretion system (Wss), flanked by a transposase, which has previously been called the Type VII secretion system [77], [78], [79], [80], [81], [82], [83]. This gene cluster has highest similarity to clusters in *E. faecalis* (25–52% amino acid sequence identity) and *Bacillus cereus* (25–53%). A putative substrate or chaperone of this Wss system, also encoded in the same gene cluster in these two *L. paracasei* strains, is a 97-residue protein with a characteristic WxG motif, no signal peptide, and highest similarity to proteins of *Clostridium acetobutylicum*, *Lactococcus lactis* ssp. *cremoris* SK11 and MG1363, streptococci and enterococci. The function of these secreted proteins is not known. In streptococci and enterococci members of this protein family are known as virulence factors, but it has been argued that a better description would be “niche-adaptation factors” [84]. The same WXG100 family protein is also encoded in two other *L. paracasei* strains (Lpp77, Lpp123) which do not appear to have the Wss system.

### Extracellular polysaccharides (EPS)

EPS biosynthesis gene clusters can be either chromosomally or plasmid-encoded. In the *L. paracasei* genomes four regions appear to represent EPS biosynthesis gene clusters (**Table 1**).

The EPS-1 region is a cassette of 7 EPS biosynthesis genes which is present in ATCC 334 (LSEI_0231–0240), BL23 and 13 other strains. Two of the genes encode rhamnosyltransferases, suggesting that rhamnose is a component of the synthesized EPS. The EPS-2 region is a cassette of 18 EPS biosynthesis genes present in strain Zhang (LCAZH_1934 -1955) and 13 other strains, but essentially absent in the other strains. The 37 *L. paracasei* strains studied here have either region EPS-1 (15 strains) or EPS-2 (14 strains or neither (8 strains); none of the strains has both EPS clusters (**Table 1**). The EPS-1 region is mainly found in Group B genomes, whereas the EPS-2 region is mainly found in Group C genomes; no correlation with sequence-based phylogeny (**Figure 2A**) or niche is evident (**Table 1**).

The EPS-3 region of ∼45 OGs (Figure****4; OG region 2950–3000) is very variable in composition, and appears to consist of 3–4 different variants of gene cassettes, each totalling about 10–20 genes. All but 8 strains (see column EPS3A in **Table 1**) have the *rmlDBCA* operon for conversion of D-glucose-1-phosphate into dTDP-L-rhamnose, suggesting that rhamnose is an important constituent of EPS in most *L. paracasei* strains. The EPS-4 region of 8 genes is only present in one strain (Lpp225), and possibly represents a teichoic acid biosynthesis gene cluster. This region is not included in the chromosomal pan-genome pseudo-assembly, as its location is unknown and could even be plasmid-located as it is on a contig with a transposase, a restriction-modification system, and toxin-antitoxin systems. Its GC content is 40% and its origin through HGT is not clear as the individual genes of EPS-4 region have best BLAST hits to various gram-positive bacteria; some encoded proteins are even 100% identical to those of phylogenetically related species *L. rhamnosus, L. buchneri* and *L. zeae*.

Taken together, it appears that strains of dairy origin in general have the least EPS biosynthesis genes, and strains isolated from plants or humans the most (**Table 1**). A few strains have very few genes for EPS biosynthesis, e.g. CNCM I-4648, Lpp71 and Lpp122; the latter 2 genomes are highly related (**Figure 2**).

### Transporters

Some ABC transporter cassettes are found to be quite variable in the 37 *L. paracasei* genomes, and this could be related to phylogenetic distance, niche adaptation and/or growth requirements:

A taurine ABC transporter (9 gene cassette), is present in strain Zhang and 11 other strains; these strains basically all belong to Group C (see **Figure 2B**) and are therefore related in genome content and presumably have a common ancestor which acquired this cassette for taurine uptake. However, the strains originate from many different niches.
One glycine betaine ABC transporter (5 gene cassette), is present only in strain Zhang and 2 other strains (Lpp46, Lpp126). There are several other highly conserved glycine betaine ABC transporters encoded in all genomes.
A ferric ion ABC transporter (5 gene cassette), directly linked to a two-component regulatory system, is present in 13 strains at the same chromosomal location, but not in the three reference genomes. There does not appear to be any relationship between these strains, as they come from both plant, dairy and human environments. This ferric ion ABC transporter is not found in any other LAB, and its closest homologs (48–84% identity) are in a gene cluster of Enterococcus italicus and some streptococci.
A cobalt/nickel ECF transporter (4-gene cassette), is present only in the unrelated strains Lpp226 and Lpp41. This transporter is on a larger fragment that is 99–100% identical to L. plantarum (see above).
A specific peptide ABC transporter (4 gene cassette) is present only in strain BL23 and 5 other strains, mainly from Group B genomes. Some of the strains cluster together, i.e. Lpp189/Lpp228 and Lpp37/Lpp223/BL23 (**Figure 2B**). These encoded proteins have 94–100% identity to *L. rhamnosus*. The significance is not clear, as there are also several oligopeptide ABC transporters and antimicrobial peptide transporters in all genomes.

Very few of these variable ABC transporters appear to be correlated with niche adaptation, contrary to what may be expected.

### Gene trait matching (GTM)

#### Sugar metabolism

**Figure 6:**
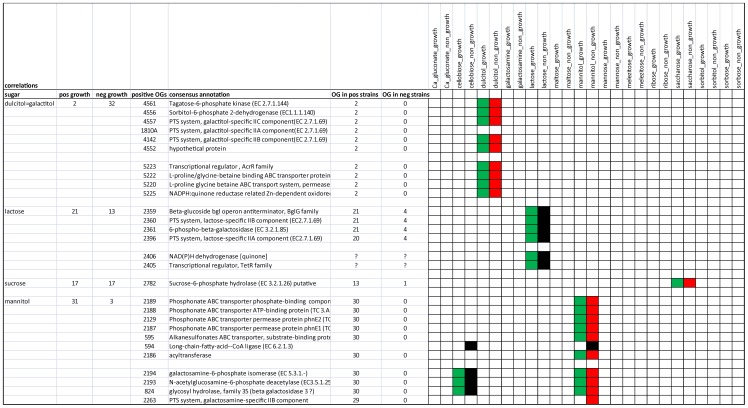
Example of the GTM output. The first column lists the sugar tested, and the second and third columns indicate the number of strains that grow (positive) or do not grow (negative) on that sugar. Relevant OGs and their annotation are listed in columns four and five. All coloured cells indicate OGs important for the classification of the specified phenotype (at top). Green cells indicate presence of the OG (>75%), red indicates absence of the OG (>75%). OGs that are important for the classification of the phenotype but are not present or absent in a large fraction of the strains are coloured black.

Among the growth profiles on different carbon sources, several results were obtained that demonstrated the power of the GTM method (details of GTM results can be found in **Table S6**). In growth studies on lactose, saccharose ( = sucrose), galactitol, mannitol, cellobiose, ribose, sorbitol and sorbose we observed matches with genes clearly involved in sugar metabolism (**Table 3** **,** **Figure 6**)**.** In some cases, these genes were even specifically annotated as involved in the breakdown or uptake of the specific sugar. A good example is a 4-gene lactose PTS cassette which is absolutely required for growth on lactose. Apparently none of the many other PTS systems can utilize lactose. The contig information indicates that this lactose gene cluster is generally on the chromosome, but in some strains could be on a plasmid.

**Table 3:** Overview of significant gene-trait matching results corresponding to growth/no growth of 34 strains in the presence of different sugars.

sugar	strains growth	strains no growth	regions, OG functions
lactose	21	13	*4-gene cassette, includes:*
			> lactose PTS transport system
			>6-phospho-beta-galactosidase
			> beta-glucoside bgl operon antiterminator
saccharose ( = sucrose)	17	17	> sucrose-6-phosphate hydrolase
galactitol ( = dulcitol)	2	32	*6-gene cassette, includes:*
			> galactitol PTS
			> tagatose-6-phosphate kinase
			> sorbitol-6-phosphate 2-dehydrogenase
			*4-gene cassette, includes:*
			> L-proline/glycine-betaine ABC transporter
mannitol	31	3	*4-gene cassette, includes:*
			> galactosamine PTS
			> galactosamine-6-phosphate isomerase
			> N-acetylglucosamine-6-phosphate deacetylase
			> glycosyl hydrolase, family 35 (beta galactosidase 3?)
			*7-gene cassette, includes:*
			> phosphonate/sulfonate ABC transporters
cellobiose	29	5	*large parts of sugar islands A and B, includes:*
			> alpha-glucosides PTS
			> maltose-6′-phosphate glucosidase
ribose	26	8	*parts of sugar islands A and B, includes:*
			> part of ribose utilization operon
			> alpha-glucosides PTS
			> maltose-6′-phosphate glucosidase
sorbitol ( = glucitol)	19	15	*large parts of sugar islands A and B, includes:*
			> galactitol PTS
			> galactosamine PTS
			> ascorbate PTS
			> cellobiose PTS
			> fructose/mannitol PTS
sorbose	21	13	*large parts of sugar island A, includes:*
			> sorbose PTS
			> many other sugar PTS

Only 2 strains grow on galactitol, which correlates with the presence of a 6-gene galactitol PTS cassette in sugar island A, corresponding to genes LCAZH_0323–0328 of *L. casei* strain Zhang. This is remarkable, since sugar island B contains 6 cassettes annotated as galactitol PTS and hence it would appear that either this annotation is incorrect or the cassettes are all inactive under the conditions tested, possibly due to strict regulation.

Growth on mannitol does not correlate with the 5 cassettes annotated as fructose/mannitol PTS in sugar island B, but does correlate with a 4-gene putative galactosamine PTS cassette, and also with a putative phosphonate ABC transporter. Lack of growth on the sugars cellobiose, ribose, sorbitol and sorbose correlates with the absence of large parts of the sugar islands A and/or B, and in general the lacking parts include cassettes annotated to be specific for those sugars (see **Figure 5** and **Table 3**; details in **Table S6**).

No specific genes were found to be significantly correlated with growth on gluconate, melezitose, galactosamine, maltose or mannose, even though some sugar utilization cassettes are annotated as being specific for galactosamine, maltose and mannose. For mannose this is perhaps not surprising, since at least 12 cassettes are annotated as mannose PTS, and hence there is a great redundancy of mannose utilization cassettes. The only two strains that do not grow on mannose lack the majority of these putative mannose PTS systems, but not all.

These results validate the use of Phenolink for matching gene presence and absence to phenotypic properties, and clearly provides many leads for improving annotation of genes.

#### Origin of isolation

We also performed a GTM analysis using the origin of each strain as input to Phenolink. The Random Forrest algorithm was able to classify the 16 strains of dairy and 10 strains of plant origin very well, but not the 8 strains of human/animal origin (data not shown). The latter are mostly fecal strains of which the origin is rather dubious as they can originate from ingested food. However, no specific genes or gene clusters were found to correlate with strain origin of isolation.

## Discussion

Our study is one of the first reports of a large number of draft genome sequences of *Lactobacillus* strains of the same species (*L. paracasei*) originating from different niches, including dairy, plant and human isolates. Comparative genomics has allowed the identification of many new genes and gene clusters compared to previously published (reference) *L. paracasei* genomes.

The size of the core genome is found to be roughly proportional to the total size of a single reference genome of each LAB species, i.e. for *S. thermophilus* (core genes 1271/total genes1900; n = 47) [85], *L. lactis* (1268/2300; n = 39) [66], *O. oeni* (1591/1691; n = 10) [28], *L. plantarum* (2049/2956; n = 42) [86], *L. sakei* (1449/1879; n = 18), *L. reuteri* (1463/2170; n = 57) [87], *L. salivarius* (1668/2184 ; n = 33 strains) [88] and *L. casei* (1941/2678; n = 21) [19].

A very interesting component of the core *L. paracasei* genome is an 8-gene cassette which encodes biosynthesis of short branched-chain fatty acids. Four genes of this cluster (*bkdABCD*) encode the branched-chain α-keto acid dehydrogenase (BKDH) complex that converts the BCKA into the corresponding branched-chain acyl-coenzyme A's by an oxidative decarboxylation. These molecules are then transformed into free fatty acids (e.g.isobutyrate, isovalerate) by two successive reactions catalyzed by the phosphobutyrylase (Ptb) and the butyrate kinase (Buk). Ptb exhibits a broad substrate specificity [61] leading to various C2 to C8 acylphosphate derivatives. These branched-chain short fatty acids are known to have a beneficial role on the preservation of the integrity of the colonic epithelium and that are associated with many biological properties in the healthy or diseased gut [89], [90]. These include the maintenance of epithelial integrity, inhibition of inflammation and modulation of energy metabolism [91]and prevention of oxidative damage to colon cells [92]. In *L. paracasei* this pathway may allow the generation of ATP from amino acid metabolism under anaerobic conditions. Hence it can give these microbes a “fitness advantage” in protein-rich anaerobic environments. Among all sequenced Lactobacillus strains to date, *L. paracasei* is the only species containing the *bkd* genes allowing the production of branched-chain fatty acids from BCKA. The genomic context around the *bkd* operon is conserved (**Figure 3a**) in other lactobacilli, which strongly suggests that the *bkd* operon had been horizontally acquired in *L. paracasei*. However, this presumably happened a very long time ago, since all *L. paracasei* strains carry this operon, and since the 49–50% GC content of this operon is only slightly higher than the *L. paracasei* genome average of 46.5%, and much higher than the 40% GC content of the *E. faecalis* operon.

Our present study now also provides a first insight in the core and pan-genome of a *Lactobacillus* species, and its variome or flexible gene pool. The core genome estimated from the full genome sequences of 37 *L. paracasei* strains is about 1800 OGs (or gene families), slightly lower than the estimate by CGH [19]. The fact that this core genome contains about 270 gene families of unknown function indicates that there is still much to be learned about the functions of highly conserved and possibly essential genes in *L. paracasei*, and this probably also applies to large genomes of other LAB such as *L. plantarum, L. pentosus* and *L. brevis*. The flexible gene pool is estimated to be at least 2400 OGs, and it appears to level off as more genomes are added (**Figure 1**). The gene families of the *L. paracasei* variome can be roughly broken down into phages (370 OGs; 15%), plasmids (230 OGs; 10%), transposon/conjugative element (180 OGs; 7%), sugar utilization cassettes (320 OGs; 13%), hypothetical proteins (880 OGs; 37%) and other known functions (420 OGs; 18%); the latter group contains e.g. cell-surface proteins, transporters, CRISPR-associated proteins (Cas), EPS biosynthesis proteins, transposases of IS elements, etc. The number of hypothetical protein families of unknown function is actually more than 1200 OGs (50% of the variome), as the categories phages, plasmids and transposon also contain numerous hypothetical proteins.

The presence and size of plasmids is rather difficult to determine from draft genomes, as plasmids generally contain many IS elements and hence the assembled contigs rarely represent complete plasmids. Nevertheless, we applied several criteria to identify putative plasmids, and conclude that the variome appears to contain many different plasmids, some larger than 20 kb (**Tables S3** and **S4**), and an integrative conjugative element which could consist of plasmid fragments inserted into the chromosome. *Lactococcus lactis* is also known to harbour a large variety of plasmids, with functions related to transport, heavy metal resistance, proteolysis, lactose utilization and EPS biosynthesis [66]. Much less is known about plasmids in lactobacilli, and few are described in the NCBI database (http://www.ncbi.nlm.nih.gov/genome/?term=lactobacillus%20plasmid). *L. paracasei* strains are known to contain larger plasmids (**Table S1**) and we found fragments similar to these plasmids in many genomes of this study (**Table S3**). *L. rhamnosus* Lc705 has a 64-kb plasmid which resembles the conjugative element in our *L. paracasei* genomes.

It has been shown in several other lactic acid bacteria, e.g. *L. plantarum* [20], [86], *L. casei* [19], *Oenococcus oeni* [28] and *Lactococcus lactis* [66], that genes for sugar uptake and metabolism can be very variable between strains. These genes are generally organized in gene cassettes or modules, encoding a transporter (1–4 subunits), a regulator, and enzymes for sugar breakdown [20], with each cassette (semi)-specific for a certain sugar. The presence or absence of entire cassettes can be highly variable, and is presumed to reflect adaptation to growth on particular sugar substrates in different niches. Often many sugar metabolism cassettes are clustered together on the chromosome, in so-called lifestyle or sugar islands [66], [86], [93]. Deviating base compositions of these genomic islands and cassettes suggest they may have been acquired through horizontal gene transfer (HGT) [94].

In the *L. paracasei* pan-genome we identified 74 sugar utilization cassettes, including 63 PTS-based cassettes, of which many are localized in two large genomic islands (**Figure 5**). The number of sugar cassettes varies enormously between *L. paracasei* strains, as 25–53 total cassettes were found per strain, of which 18–44 PTS systems. Most strains contain 35 or more PTS systems, which is considerably higher than reported for other LAB such as *L. pentosus*, *L. johnsonii, L. acidophilus, L. salivarius* and *L. plantarum* with 16, 16, 20, 23 and 25 PTS [93], [95], [96], [97], [98]. However, the latter numbers refer to single genomes of each species, and it is quite likely that higher numbers of PTS systems may be found upon sequencing of more strains of each LAB species. Nevertheless, several important conclusions about sugar utilization can be drawn from our *L. paracasei* pan-genome analysis. First, *L. paracasei* appears to be highly adapted to growth on a large variety of carbohydrates. Second, there are at least 15 sugar cassettes which occur in all strains investigated and hence belong to the core genome of *L. paracasei*. Third, there are large differences between the types and number of sugar utilization cassettes, and most of the variable cassettes are organized in two large genomic islands, or “sugar life-style” islands. Fourth, a subgroup of strains of mainly dairy origin (Group A in **Figure 2B**), has significantly lower numbers of PTS cassettes, i.e. 18–30 (**Figure 5**), as also observed in the CGH study of *L. casei* strains [19], possibly reflecting the fact that in the rich dairy niches there is no longer the necessity to maintain uptake systems for a large variety of different carbohydrates. Several reports have shown that adaptation to the dairy niche can be associated with a major deletion and inactivation of genes which are no longer required for the rich dairy environment. Examples are *Streptococcus thermophilus*, *L. delbrueckii* ssp. *bulgaricus* and *L. helveticus*, organisms used in yogurt and cheese manufacture which have suffered massive gene decay leading to loss of functions in carbohydrate metabolism, amino acid and cofactor biosynthesis [99], [100], [101], [102]. This large inter-strain diversity of sugar utilization cassettes presumably applies to most other LAB, and has been substantiated for *L. plantarum* by CGH and genome sequencing [20], [86], and for *O. oeni* by genome sequencing [28].

The evolution, relatedness and niche adaptation of *L. paracasei* strains appears to be a complex story. The phylogenetic tree based on sequence similarity of core genes (**Figure 2A**) is not the same as the phylogenomic tree based on total genome contents (**Figure 2B**), albeit that there are sets of strains that cluster closely together in both trees, suggesting that these are closely related in evolution, e.g. Lpl7/Lpl14, Lpp48/Lpp225, Lpp7/Lpp71/Lpp122, Lpp46/Zhang, Lpp74/Lpp27, Lpp223/BL23, etc. However, in many cases these closely related strains have been isolated from completely different niches (**Table 1**), which casts some doubt on the validity of niche/isolation source of some of these strains. A similar observation was made in a strain diversity study of *L. plantarum*, where the human isolates (generally from faeces) were mostly scattered throughout the phylogenomic tree, suggesting that they originated from the food eaten by the individuals [86].

In the phylogenomic tree (**Figure 2B**), three major groups A-B-C can be distinguished which show clear differences in genome content relating to e.g. CRISPRs, sugar utilization cassettes, EPS biosynthesis genes, but again only group A is mainly of dairy origin, and groups B and C are of mixed origin. This indicates that the relationships between niche adaptation and phylogenetic clusters is not simple, suggesting ecological flexibility/diversity within these groups. A common loss of genes is evident in the dairy strains of group A, but acquisition of genes and gene clusters by HGT is also evident in some branches or sub-branches. There are examples of early acquisition of gene clusters, which are present in a larger set of strains and have a GC content reasonably close to that of the *L. paracasei* chromosome, and such genes are often also present in related species such as *L. rhamnosus* and *L. zeae*. But there are also examples of recent acquisition of gene clusters present in only a few related strains, where the GC content deviates much more, suggesting recent HGT.

We hypothesized that the ability to utilize carbon sources as a growth substrate is likely to be an important fitness driver in the different niches that are inhabited by *L. paracasei.* We therefore refined our niche-adaptation analysis by trying to establish correlations between sugar utilization gene (cassette) presence/absence and isolation source. Strains were divided in three different groups: strains from plant origin, mammalian origin and dairy origin. Our strain set contained a total of 16 strains that were isolated from dairy substrates. It is perhaps important to note that five of these strains originated from commercial dairy products, and the inoculating strain may have been from a completely different origin, e.g. human gut. Eleven strains originate from artisanal dairy products from countries where the fermented milk products are homemade and are produced by re-inoculating with the same strains. These are relatively “open” ecosystems and hence they may be subject to entry of strains from non-dairy origin. Our results clearly demonstrate that 7 of these dairy strains cluster together (in Group A) and contain a reduced number of sugar cassettes (33 average, see **Figure 5**) and a reduced genome size (2.8 Mb average). The “dairy niche” is characterized by a very limited spectrum of carbohydrates, where lactose is the predominant sugar. Hence, “genome reduction” by eliminating sugar cassettes for plant-derived sugars might be an effective strategy to optimize fitness. No clear genomic signatures could be identified for plant and mammal-derived isolates. This may be explained by at least two reasons. Firstly, the plant-derived isolates do not originate from a single homogenous niche but rather from an array of niches that may differ largely in sugar content and physico-chemical conditions, making it unlikely that a single optimized genomotype exists. Mammalian isolates mostly originated from the gut which is an open ecosystem that is continuously exposed to food-derived transiting microbes that may originate from dairy or plant-based products, which interferes with the identification of mammal-niche genomotypes [86].

The gene-trait matching (GTM) experiments have clearly demonstrated that genes or gene clusters (cassettes) can be identified which correlate with growth or non-growth on specific sugars. In several cases the present annotation of genes in cassettes (e.g. PTS) did not agree with the observed phenotype (**Tables 3** and **S6**), suggesting that the annotation may be wrong, too specific or too broad. The GTM results obtained for growth on sugars has provided a proof-of-principle for the Phenolink gene-trait matching protocol, and provide clear leads for validation of the gene-trait relations. However, the causality of correlations between the identified genes and the observed sugar utilization phenotypes has not been proven yet. Additional experiments such as knocking out target genes, or over-expressing the genes in strains that lack the phenotype, will increase the understanding of the type of relation between the target gene and phenotype. This observation increases the likelihood that GTM will also identify valid leads for mechanistic understanding in other, more complex phenotypes, also in cases where the link between the phenotype and the matching genes is less clear. In addition these genes can be used for the large-scale screening of culture collections for the presence of strains with the desired phenotype, thereby increasing the value and diversity of the strains in the culture collection.

There is also a clear advantage of genome sequencing as a basis for gene-trait matching (GTM) in comparison to comparative genome hybridization (CGH) data. Since the microarray platform used for CGH is often based on the genome sequence of a single strain, one would only find presence/absence patterns of the OGs for which a representative is present in the strain used for the array design. In case of *L. paracasei,* if the ATCC 334 reference strain was used for the microarray design, one would miss information on a large number of pan-genome genes, which could have a big effect on the GTM results.

Finally, our pan-genome analysis of *L. paracasei* strains has also provided a first insight in the variability of many cell-surface proteins and exopolysaccharides (**Tables 1** and **2**). Some of these extracellular proteins are found to be fully conserved, belonging to the core genome, whereas other extracellular proteins and exopolysaccharides are highly variable, occurring in only a limited number of strains. In general, strains of dairy origin have the least EPS biosynthesis genes, and strains isolated from plants or humans the most. The finding that several factors that have been associated with host-microbe interactions such as pili, the cell-envelope proteinase PrtP or proteins Msp2/p40 and Msp1/p75 have been found in all analysed *L. paracasei* strains suggests that those factors are not exclusively responsible for the specific interaction of one strain with host cells. These factors belonging to the core genome of the *L. paracasei* species are probably part of the complex machinery involved in the cross talk between bacterial strains and human or animal gut.

## Supporting Information

Figure S1 **Comparison of MLST and CRISPR typing of strains.** Neighbor-joining tree based on seven MLST gene sequences (*fusA, ileS, leuS, lepA, pyrG, recA, recG)* used previously [13]. Sequence type (ST) and CRISPR type (CT) are given for each strain. CT numbers −1 and 0 designate strains with no PCR amplification and strains with no CRISPR locus, respectively. Red dots indicate strains which were selected for genome sequencing.
(PPTX)

Table S1 **Reference** ***L. (para)casei*** **strains and genomes.**
(DOCX)

Table S2 **Statistics of** ***L. paracasei*** **genome sequencing.**
(XLSX)

Table S3 **Examples of putative** ***Lactobacillus paracasei*** **plasmids (or their fragments).**
(DOCX)

Table S4 **Encoded functions on (putative) plasmids and on the putative inserted plasmid/transposon region.**
(DOCX)

Table S5 **CRISPR loci variants. A/ Lcas1 sequences of the direct repeats and spacers, B/ Lcas2 sequences of the direct repeats and spacers.**
(XLSX)

Table S6 **Overview of significant gene-trait matching results corresponding to growth of strains in the presence of different sugars.**
(XLSX)

Supporting Information S1 **CRISPR analysis.**
(DOCX)
