# Supplementary figures and images for: Lactobacillus paracasei Comparative Genomics: Towards Species Pan-Genome Definition and Exploitation of Diversity
Source: PLoS One. 2013 Jul 19;8(7):e68731. doi: 10.1371/journal.pone.0068731 (PMC3716772; doi:10.1371/journal.pone.0068731)

## Slide 1
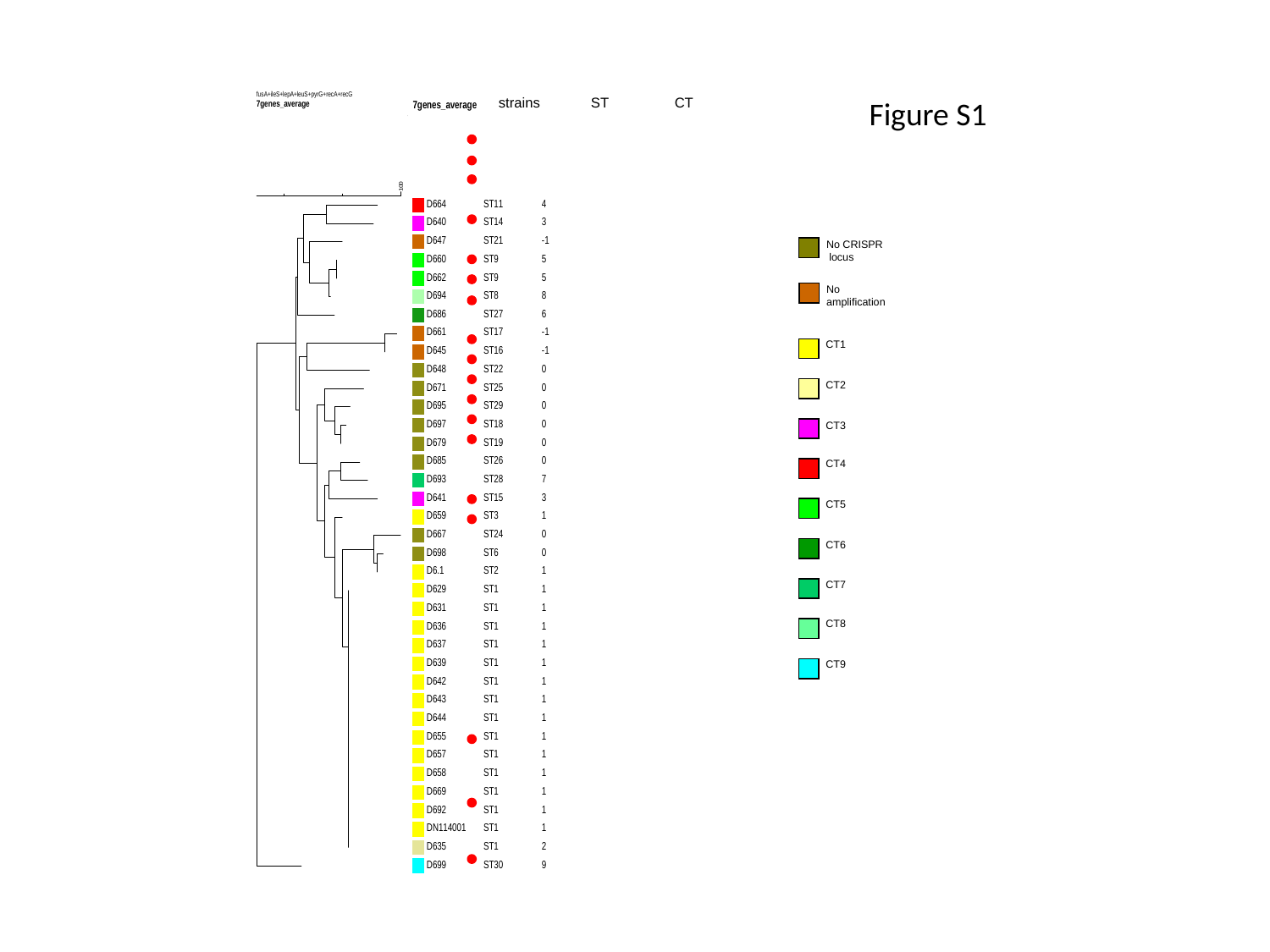

strains
 ST
 CT
Figure S1
No CRISPR
 locus
No
amplification
CT1
CT2
CT3
CT4
CT5
CT6
CT7
CT8
CT9

Supplement: Figure S1 — Comparison of MLST and CRISPR typing of strains. Neighbor-joining tree based on seven MLST gene sequences (fusA, ileS, leuS, lepA, pyrG, recA, recG) used previously [13]. Sequence type (ST) and CRISPR type (CT) are given for each strain. CT numbers −1 and 0 designate strains with no PCR amplification and strains with no CRISPR locus, respectively. Red dots indicate strains which were selected for genome sequencing. (PPTX) [file pone.0068731.s001.pptx]
