# Supplementary material for: Lactobacillus paracasei Comparative Genomics: Towards Species Pan-Genome Definition and Exploitation of Diversity
Source: PLoS One. 2013 Jul 19;8(7):e68731. doi: 10.1371/journal.pone.0068731 (PMC3716772; doi:10.1371/journal.pone.0068731)
Supplement: Table S1 — Reference L. (para)casei strains and genomes. (DOCX) [file pone.0068731.s002.docx]

**Table S1: Reference *L. (para)casei* strains and genomes**

| **species** | **strain** | **origin** | **chromosome**  **(Mb)** | **contigs** | **GC%** | **Protein ORFs** | **plasmids** | **reference** |
| --- | --- | --- | --- | --- | --- | --- | --- | --- |
| *L. casei* | ATCC 334 | cheese | 2.895 | 1 | 46.6 | 2748 | p1 (29.1 kb) | [[1](#_ENREF_1)] |
| *L. casei* | BL23 | cheese | 3.079 | 1 | 46.3 | 3015 | none | [[2](#_ENREF_2)] |
| *L. casei* | Zhang | koumiss | 2.862 | 1 | 46.5 | 2804 | plca36 (36.5 kb) | [[3](#_ENREF_3)] |
| *L. casei* | BD-II | koumiss | 3.070 | 1 | 46.3 | 3139 | pBD-II (57.4 kb) | [[4](#_ENREF_4)] |
| *L. casei* | LC2W | traditional dairy product | 3.039 | 1 | 46.4 | 3121 | pLC2W (38.4 kb) | [[5](#_ENREF_5)] |
| *L. paracasei* | 8700:2 | human gastro-intestinal tract | 2.978 | 90 | 46.2 | 3021 | n.d. | Antonsson et al, 2010, unpublished; NZ_ABQV01000000 |
| *L. paracasei* | ATTC25302 | human gastro-intestinal tract | 2.886 | 174 | 46.5 | 3042 | n.d. | Qin et al, 2009, unpublished; NZ_ACGY01000000 |

n.d. = not determined

1. Makarova K, Slesarev A, Wolf Y, Sorokin A, Mirkin B, Koonin E, Pavlov A, Pavlova N, Karamychev V, Polouchine N *et al*: **Comparative genomics of the lactic acid bacteria**. *Proc Natl Acad Sci U S A* 2006, **103**(42):15611-15616.

2. Maze A, Boel G, Zuniga M, Bourand A, Loux V, Yebra MJ, Monedero V, Correia K, Jacques N, Beaufils S *et al*: **Complete genome sequence of the probiotic Lactobacillus casei strain BL23**. *J Bacteriol* 2010, **192**(10):2647-2648.

3. Zhang W, Yu D, Sun Z, Wu R, Chen X, Chen W, Meng H, Hu S, Zhang H: **Complete genome sequence of Lactobacillus casei Zhang, a new probiotic strain isolated from traditional homemade koumiss in Inner Mongolia, China**. *J Bacteriol* 2010, **192**(19):5268-5269.

4. Ai L, Chen C, Zhou F, Wang L, Zhang H, Chen W, Guo B: **Complete genome sequence of the probiotic strain Lactobacillus casei BD-II**. *J Bacteriol* 2011, **193**(12):3160-3161.

5. Chen C, Ai L, Zhou F, Wang L, Zhang H, Chen W, Guo B: **Complete genome sequence of the probiotic bacterium Lactobacillus casei LC2W**. *J Bacteriol* 2011, **193**(13):3419-3420.
