# Supplementary material for: Lactobacillus paracasei Comparative Genomics: Towards Species Pan-Genome Definition and Exploitation of Diversity
Source: PLoS One. 2013 Jul 19;8(7):e68731. doi: 10.1371/journal.pone.0068731 (PMC3716772; doi:10.1371/journal.pone.0068731)
Supplement: Table S3 — Examples of putative Lactobacillus paracasei plasmids (or their fragments). (DOCX) [file pone.0068731.s004.docx]

**Supporting Information**

**Table S3: Examples of putative *Lactobacillus paracasei* plasmids (or their fragments)^a^**

| **strain** | **plasmid/contigs** | **Size (kb)** | **plasmid type ^b^** | **similar to ^c^** |
| --- | --- | --- | --- | --- |
| Zhang | plca36 | 36.5 | A |  |
| ATCC 334 | p1 | 29.1 | B |  |
| BD-II | pBDII | 57.4 |  |  |
| LC2W | pLC2W | 38.4 |  |  |
|  |  |  |  |  |
| Lpp7 | c0165 | 5.9 | K | pLC2W |
| Lpp14 | c0306 | 5.7 |  | pLC2W, pLC1 |
| Lpp17 | c0080  c0114  c0164  c0173 | 8.9  7.4  5.8  12 | H  K  F | pLC2W, pLC1 |
| Lpp22 | c0019  c0031  c0143/c0133  c0278  c0279 | 11.5  5.8  5.7  3.8  3.3 | F  G | pLC2W, pBDII, pLC1 |
| CNCM I4648 | c0005  c0683  c0709 | 20  5.2  5.4 | A  E  K | plca36  pLC2W, p1, pLC1 |
| Lpp41 | c0055  c0109  c1007 | 5.8  5.7  4.9 | F  K  K | pLC2W, pBDII |
| Lpp43 | c0012  c0138 | 52  3.8 | A  D | plca36 |
| Lpp46 | c0016 | 7.6 | D |  |
| Lpp48 | c0018/c0019  c0793 | 4.5  2.9 | C |  |
| Lpp49 | c0067 | 5.3 | K | pLC2W, pLC1 |
| Lpp70 | C0081 | 9.3 |  | p1 |
| Lpp71 | c0021  c0038  c0653 | 4.5  8.9  5.2 | C  H  K | pLC2W, pLC1 |
| Lpp74 | c0058  c0097  c0098 | 16.7  5.8  5.6 |  | pLC2W, pBDII, pLC1  pLC2W, pBDII, pLC1  pLC2W, pLC1 |
| CNCM I4270 | c0011  c0462  c0517/c0536 | 4.1  9.2  6.9 | C  D | pLC2W, pBDII |
| CNCM I2877 | c0138  c0371 | 9.8  3.2 | D | pLC2W, pBDII |
| Lpp120 | c0010  c0044  c0046  c0098  c0118  c0228 | 4.5  2.5  5.1  5.3  3.4  7.3 | C  F  E  A | plca36 |
| Lpp122 | c0080  c0087  c101 | 7.4  6.2  4.9 | F  C  J |  |
| Lpp123 | c0890  c1262  c1263 | 5.8  7.4  5.1 | D  E  E |  |
| Lpp125 | c0091  c0124  c0130 | 10  4.9  4.6 | F  K | pLC2W, p1, pLC1  pLC2W, pBDII, pLC1 |
| Lpp126 | c0008  c0827 | 23  5.4 | A | plca36  pLC2W, pLC1 |
| Lpp189 | c0016/c0211  c0095  c0153 | 33  10.3  5.3 | A  K | plca36  p1  pLC2W, p1, pLC1 |
| Lpp219 | c0126  c0146 | 6.3  4.9 |  | pLC1 |
| Lpp221 | c0130  c0158 | 5.7  3.8 | G | pLC2W, pLC1 |
| Lpp223 | c0008  c0009  c0195 | 8.9  2.4  5.0 | K | pLC2W, pLC1 |
| Lpp225 | c0044 | 4.7 | C |  |
| Lpp226 | c0017 | 3.4 |  | pLC2W, pBDII, pLC1 |
| Lpp228 | c0017  c0091  c0112 | 35  10.3  6.5 | A  K | plca36  p1  pLC2W, p1, pLC1 |
| Lpl7 | c0051/c0056  c0045 | 9.6  7.4 | D  J |  |
| Lpl14 | c0092  c0079 | 5.3  7.3 | D  J |  |

No plasmids are found in strains BL23, Lpp37, Lpp230, CNCMI4649.

**^a^** Not all putative plasmid fragments are shown.

**^b^** Based on sequence similarity to each other.

**^c^** Similar to part of known plasmid (see Table 1 for references); pLC1 is found in *L. rhamnosus* Lc705
