# Supplementary material for: Lactobacillus paracasei Comparative Genomics: Towards Species Pan-Genome Definition and Exploitation of Diversity
Source: PLoS One. 2013 Jul 19;8(7):e68731. doi: 10.1371/journal.pone.0068731 (PMC3716772; doi:10.1371/journal.pone.0068731)
Supplement: Table S4 — Encoded functions on (putative) plasmids and on the putative inserted plasmid/transposon region. (DOCX) [file pone.0068731.s005.docx]

**Supporting Information**

**Table S4: Encoded functions on (putative) plasmids and the putative inserted plasmid/transposon region**

| **Encoded functions** |
| --- |
| **Plasmids**   - replication proteins - chromosome (plasmid) partitioning protein ParA - conjugations proteins (TraBCDEFHIJKL) - Site-specific recombinase, DNA invertase - type I RM system - LtrC-like (low temperature requirement) protein; phosphatidylglycerophosphatase - Toxin-antitoxin systems - PTS transporters (several) and sugar utilization enzymes - Cation-transporting ATPases - copper chaperone - Arsenate resistance - Cystathionine beta-synthase, cystathionine gamma-lyase, serine acetyltransferase - transposases (various)   **Plasmid/transposon/ICE insert region**   - replication proteins - replication initiator protein - conjugation proteins (TraE, TraG) - chromosome (plasmid) partitioning protein ParA - DNA-repair protein (SOS response UmuC-like protein) - relaxase Mob DEI - DNA/RNA non-specific endonuclease - type I RM system, restriction subunit R (EC 3.1.21.3) - site-specific recombinase, DNA invertase Pin related protein - LtrC-like (low temperature requirement) protein; phosphatidylglycerophosphatase - cell wall-associated hydrolase, NlpC/P60 family - collagen-adhesion protein - D-lactate dehydrogenase (EC 1.1.2.5) - Na+/xyloside symporter related transporter - di- and tricarboxylate transporter - Na+/H+ antiporter NapA - cation-transporting ATPase - voltage-gated chloride channel protein - PTS transporters (e.g. mannose) - fumarate reductase - transposases (various) |
