# Supplementary material for: Lactobacillus paracasei Comparative Genomics: Towards Species Pan-Genome Definition and Exploitation of Diversity
Source: PLoS One. 2013 Jul 19;8(7):e68731. doi: 10.1371/journal.pone.0068731 (PMC3716772; doi:10.1371/journal.pone.0068731)
Supplement: Supporting Information S1 — CRISPR analysis. (DOCX) [file pone.0068731.s008.docx]

**Supporting Information: CRISP analysis**

**Introduction**

Clustered regularly interspaced short palindromic repeats (CRISPRs) loci, an important phage-resistance system, have proven to be highly variable in genetically homogeneous species. Because CRISPR loci are present in many bacterial species, and because their variation can be conveniently scored either by sequencing or by DNA hybridization, they represent a promising typing tool [[1](#_ENREF_1),[2](#_ENREF_2)]. It is considered that CRISPR loci are hypervariable because rapid evolution would be driven by phage predation [[3](#_ENREF_3),[4](#_ENREF_4),[5](#_ENREF_5),[6](#_ENREF_6)]. CRISPRs are composed of short highly conserved direct repeats (DR), separated by variable spacer sequences which are derived from phages or other mobile elements. There are two CRISPR loci in *L. paracasei* strains, called Lcas1 and Lcas2, identified by the presence of characteristic CRISPR-associated (*cas*) genes. The CRISPR repeats and spacers for published genomes ATCC 334 and BL23 have been identified earlier [[5](#_ENREF_5)]. In L. casei the Lcas1 locus was identified in the genome of strain [BL23](http://www.sciencedirect.com/science?_ob=RedirectURL&_method=externObjLink&_locator=genbank&_issn=01681605&_origin=article&_zone=art_page&_plusSign=%2B&_targetURL=http%253A%252F%252Fwww.ncbi.nlm.nih.gov%252Fentrez%252Fquery.fcgi%253Fcmd%253Dsearch%2526db%253Dnucleotide%2526doptcmdl%253Dgenbank%2526term%253DBL23%5baccn%5d) but not in that of [ATCC 334](http://www.sciencedirect.com/science?_ob=RedirectURL&_method=externObjLink&_locator=genbank&_issn=01681605&_origin=article&_zone=art_page&_plusSign=%2B&_targetURL=http%253A%252F%252Fwww.ncbi.nlm.nih.gov%252Fentrez%252Fquery.fcgi%253Fcmd%253Dsearch%2526db%253Dnucleotide%2526doptcmdl%253Dgenbank%2526term%253DATCC334%5baccn%5d), whereas the Lcas2 locus present in [ATCC 334](http://www.sciencedirect.com/science?_ob=RedirectURL&_method=externObjLink&_locator=genbank&_issn=01681605&_origin=article&_zone=art_page&_plusSign=%2B&_targetURL=http%253A%252F%252Fwww.ncbi.nlm.nih.gov%252Fentrez%252Fquery.fcgi%253Fcmd%253Dsearch%2526db%253Dnucleotide%2526doptcmdl%253Dgenbank%2526term%253DATCC334%5baccn%5d) was absent in [BL23](http://www.sciencedirect.com/science?_ob=RedirectURL&_method=externObjLink&_locator=genbank&_issn=01681605&_origin=article&_zone=art_page&_plusSign=%2B&_targetURL=http%253A%252F%252Fwww.ncbi.nlm.nih.gov%252Fentrez%252Fquery.fcgi%253Fcmd%253Dsearch%2526db%253Dnucleotide%2526doptcmdl%253Dgenbank%2526term%253DBL23%5baccn%5d) [[7](#_ENREF_7)](**Table 1**).

**Methods**

We investigated the presence and diversity of CRISPR loci Lcas1 and Lcas2 in a collection of 37 *L. paracasei* strains **(Figure S1**) from the Danone Research collection, including strains BL23 (coded as D692) and type strain ATCC 334 (D671), and 16 of these strains were later selected for genome sequencing (**Table1**). In brief, two primer pairs, Pp1 and Pp3, were designed to amplify the Lcas1 region; Pp1 was designed to amplify the Lcas1 locus and contained forward primer GTCGTTCTCATCGAATTCTC and reverse primer GTGCATGCTTTCTACAATAGG. Primer pair Pp3 was designed to amplify by long-range PCR (Promega kit) a larger portion of the same region; it contained forward primer GATTCTGTCGCAATCTTATGG and reverse primer GTTCAACGCTATCATGATTGG . The amplification with pair Pp3 was used to screen for the presence of the CRISPR locus; when the locus was absent, a fragment of 3,433 bp could be amplified. Primer pair Pp4 was designed to amplify a 1,642 bp fragment of locus Lcas2 [[5](#_ENREF_5)] in strain ATCC 334 [[8](#_ENREF_8)]. The pair contained forward primer CCGCTTGTTAAGAAATCCAG) and reverse primer CTTATCAGGGACGTATAGCG. Sequencing was performed using Big Dye chemistry using PCR primers and ad-hoc internal primers. MLST data were available from the previous study as described [[9](#_ENREF_9)]. Construction of a minimum spanning tree based on allelic profiles, and neighbor-joining analysis of concatenated sequences were performed using BioNumerics v5.10 (Applied-Maths, Sint-Martens-Latem, Belgium).

**Results**

In a preliminary study, we first sequenced CRISPR loci in a collection of 37 *L. paracasei* strains and compared its diversity with MLST (**Figure S1**). A CRISPR Lcas1 locus sequence was found in 26 of 37 strains. All these loci had one typical direct repeat (DR) sequence (**Table S5A**), which was totally identical to the Lcas1 DR sequence of BL23, attributed to family Lsal1 within the 36-nt repeat supra family [[5](#_ENREF_5)]. In total 139 distinct spacers were identified, all being 30 bp in length. Nine distinct Lcas1 CRISPR sequence types could be distinguished, differing by the number and identity of CRISPR spacers as well as by single nucleotide polymorphisms (SNP) in the direct repeat spacers of 30 nt. Clearly, CRISPR sequence variation was highly concordant with MLST diversity, as strains with identical or related STs had identical or similar CRISPR types (**Figure S1**). Locus Lcas2 with a 28-bp DR and 33-bp spacers, belonging to the diverse Ldbu1 family [[1](#_ENREF_1)], was detected only in strain ATCC 334.

**References**

1. Horvath P, Romero DA, Coute-Monvoisin AC, Richards M, Deveau H, et al. (2008) Diversity, activity, and evolution of CRISPR loci in Streptococcus thermophilus. J Bacteriol 190: 1401-1412.

2. Pourcel C, Salvignol G, Vergnaud G (2005) CRISPR elements in Yersinia pestis acquire new repeats by preferential uptake of bacteriophage DNA, and provide additional tools for evolutionary studies. Microbiology 151: 653-663.

3. Barrangou R, Fremaux C, Deveau H, Richards M, Boyaval P, et al. (2007) CRISPR provides acquired resistance against viruses in prokaryotes. Science 315: 1709-1712.

4. Horvath P, Barrangou R (2010) CRISPR/Cas, the immune system of bacteria and archaea. Science 327: 167-170.

5. Horvath P, Coute-Monvoisin AC, Romero DA, Boyaval P, Fremaux C, et al. (2009) Comparative analysis of CRISPR loci in lactic acid bacteria genomes. Int J Food Microbiol 131: 62-70.

6. Sorek R, Kunin V, Hugenholtz P (2008) CRISPR--a widespread system that provides acquired resistance against phages in bacteria and archaea. Nat Rev Microbiol 6: 181-186.

7. Maze A, Boel G, Zuniga M, Bourand A, Loux V, et al. (2010) Complete genome sequence of the probiotic Lactobacillus casei strain BL23. J Bacteriol 192: 2647-2648.

8. Makarova K, Slesarev A, Wolf Y, Sorokin A, Mirkin B, et al. (2006) Comparative genomics of the lactic acid bacteria. Proc Natl Acad Sci U S A 103: 15611-15616.

9. Diancourt L, Passet V, Chervaux C, Garault P, Smokvina T, et al. (2007) Multilocus sequence typing of Lactobacillus casei reveals a clonal population structure with low levels of homologous recombination. Appl Environ Microbiol 73: 6601-6611.
